# Improving the torsion performance of IPMC by changing the electrode separation

**DOI:** 10.1038/s41598-021-87347-z

**Published:** 2021-04-07

**Authors:** Bing Xu, ShiHu Wang, ZiFeng Zhang, Jing Ling, XinTao Wu

**Affiliations:** 1 Department of Mechanical Engineering, Chao Hu University, Hefei, China; 2 Department of Robotics Engineering, Yangtze Normal University, Chongqing, China

**Keywords:** Soft materials, Structural materials, Structural biology

## Abstract

Ionic polymer metal composites (IPMCs) are widely studied as actuators and sensors, due to their large bending motion, flexibility and being light-weight. Nowadays, IPMCs are used in the bionic field, for example, to achieve bending and twisting movements of wings and fins. In this paper, a method is proposed to optimize the torsion performance of IPMCs by changing the electrode separation. The IPMCs with patterned electrode fabricated by masking technique are proposed to accomplish twisting motion. The result indicates that the torsion performance is improved as the electrode separation increased. Thereby it provides a new strategy for the bionic field with twisting behavior.

## Introduction

IPMC is a typical electro-active polymer with a ‘sandwich’ structure, which comprised by an ion exchange membrane and two thin metal electrode layers. It is proposed to be used in the area of aerospace, space technology, bionic machinery, bio-medical treatment, micro-actuator and energy storage for its flexibility, lightness, low driving voltage and large bending motion^1–7^. Nowadays IPMC have been studied as actuators, such as micro-grippers^8^, underwater robots^9^, micro-pumps^10–12^, bionic fish^13,14^, dragonflies^15^ and butterflies^16^, focusing on the large bending motion under low voltage. Their characteristic of large bending motion is generated by redistribution of its internal water molecules under low voltage. When the electric field is applied across the thickness of an IPMC, the hydrated cations with water molecules move together towards the cathode which results in swelling near the cathode side and contraction near the anode side of the IPMC. The internal extensional stress of the polymer causes the IPMC to bend towards the anode direction^17–20^.

**Figure 1:**
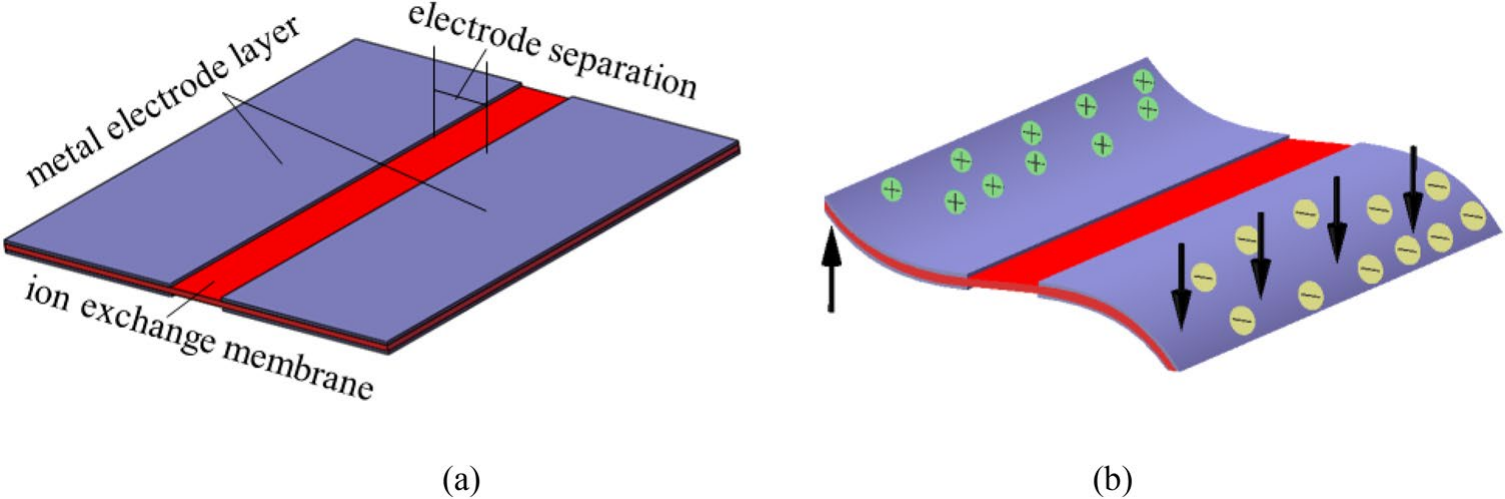
The twisting motion of IPMC with patterned electrode. (**a**) Before applying voltage. (**b**) After applying voltage.

It is well known that creatures in nature often move with multiple degrees of freedom. In order to imitate this complex movement, some researchers have carried out complex movements that can be achieved by making different types of IPMC. Jeon et al. used masking technology to obtain an IPMC, which can achieve multi-segment motion, and the torsion angle can reach 3 degrees under a voltage of 1 V^21^. Riddle et al. obtained IPMC with patterned electrodes through micromachining, which can obtain bending and twisting motions at the same time. The torsion angle can exceed 7 degrees^22^. Rossiter et al. use hot-melt technique and electrical discharge machining to get an IPMC with patterned electrodes by wiping off the excess electrode^23^. Nakabo et al. fabricated a multi-degree manipulator using IPMC with patterned electrodes. The manipulator can realize twisting motion by applying a voltage to the IPMC, the experimental and simulation results show that the torsion angle can reach 4 degrees^24^. Feng et al. presents a novel helical ionic polymer metal composite spring actuator, which can realize helical motion drive by a square wave^25^. Hubbard et al. used a simple surface processing technology to create IPMC with unique patterned electrodes, which can achieve bending, twisting, flapping and other bio-inspired locomotive behaviors. The experimental results show that the twist angle exceeded 8 degrees^26^. Palmre et al. designed bio-inspired active fins for underwater application, which were fabricated by embedding the molding IPMC actuators into soft materials. The fins can realize bending motion and twisting motion underwater, and the twist angle was measured exceeded 10 degrees^13^. Shin et al. used a commercial milling machine to process an electrode layer with controllable thickness and pattern on the surface of IPMC, and the actuator produced undulating motion as expected^27^. Chang proposed a novel actuating structure of IPMC with single layered electrodes, in which the electrodes were fabricated on one side of the substrate, leading to distinct S-shaped deformation with large displacement and high load capacity^28^. These researchers aim to use different methods to manufacture IPMCs, such as masking technology, micromachining, in order to get better torsion performance. In this paper, IPMC with patterned electrodes was fabricated by electroless chemical reduction. When the voltage applied on different parts of electrode, IPMC will generate complex motion, as shown in Fig. 1. We have investigated the effect of electrode separation of IPMC on torsion performance. The twisting motion was recorded by a digital camera (SONY Alpha 6400) under AC voltage drive, and the results were discussed in the remainder of this paper.

## Experiment

### Fabrication of IPMC with patterned electrodes

Materials used in the experiment are shown in Table 1. The metal layer of IPMC is platinum.

**Table 1:** Materials used in the experiment.

Name	Molarity/concentration/thickness	Manufacturer
Nafion film	178 μm	Dupont China
[Pt(NH_3_)_4_]Cl_2_ powder	58%	Shanghai Aladdin Biochemical Technology Co., Ltd
H_2_SO_4_	0.5%	Hefei Zhongwan Chemical Reagent Co., Ltd
H_2_O_2_	15%	
NaBH_4_	5%	
Hydrazine hydrate	20%	
Hydroxylammonium chloride	5%	
Ammonium hydroxide	5%	
LiCl solution	1 mol/L	
De-ionized water	–	Chao Hu University

The literature^21^ mentioned that IPMC with patterned electrodes prepared by mask technology. First, the Nafion membrane is covered with masking tapes, and then the IPMC with patterned electrodes is fabricated by electroless chemical reduction. The concrete steps are as follows.

First of all, the making tapes (polyimide membrane) should be paste on both sides of Nafion membrane to protect it without metallization. In order to increase the interfacial area, 1600# sandpaper was used to roughen the surface of Nafion membrane, which helps more platinum particles penetrating into the membrane. Second, the membrane was cleaned with H_2_SO_4_ (0.5%) and H_2_O_2_ (15%) solutions for 1 h respectively, and cleaned with de-ionized water at 100 °C for 1 h. And then, immersed the membrane in a solution of [Pt(NH3)4] Cl2 (3 mg/mm^2^) for about 14 h to accomplish the ion exchange. Third, in order to metalize platinum particles on the surface of the membrane, the reducing agent NaBH_4_ (5%) was added to the solution. Two hours later, the metal layers were formed. Next, before the second reduction reaction, the membrane was cleaned in an ultrasonic cleaner for half an hour. Forth, hydrazine hydrate solution (20%) and hydroxylammonium chloride solution (5%) were added to the mixed solution of tetraammineplatinum chloride hydrate and 5% ammonium hydroxide to complete the reduction reaction. The reaction temperature was varied from 40 to 60 °C. Finally, the IPMC samples were washed with deionized water and stored in LiCl solution (1 mol/L).

### Experimental setup

**Figure 2:**
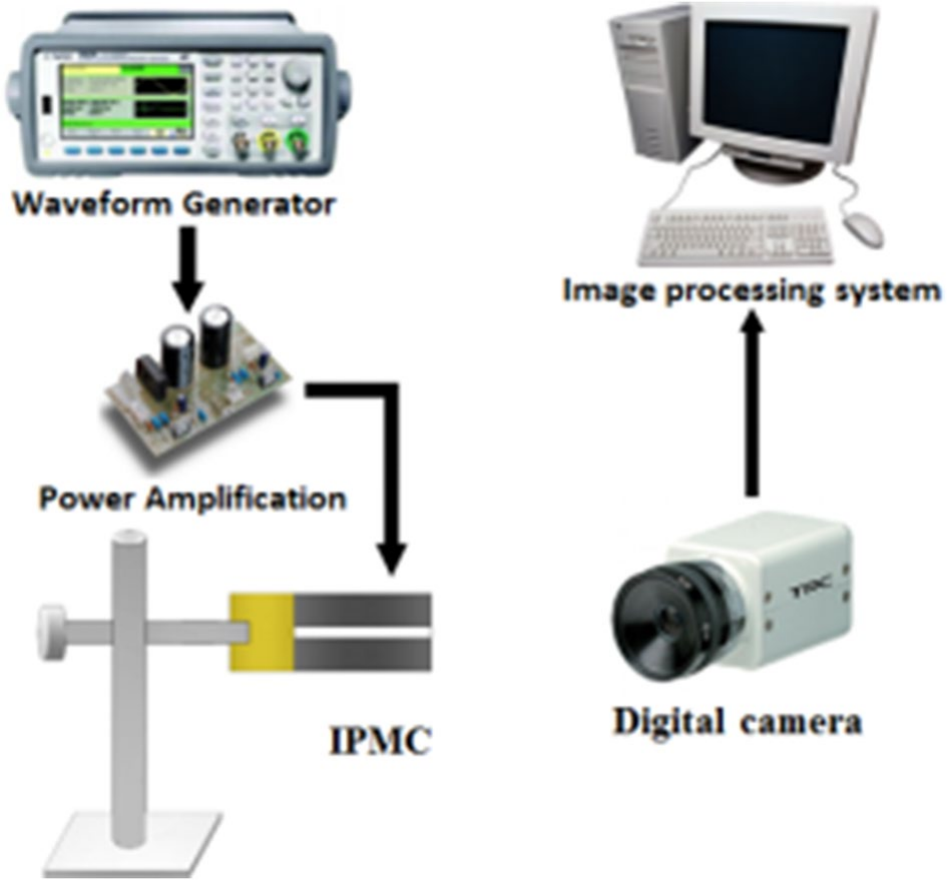
Torsion performance test system.

Twist angle is the main performance parameter of IPMC with patterned electrodes. The experimental setup of the torsion performance test system is shown in Fig. 2. Instruments used in the test are shown in Table 2. The IPMC was gripped above the ruler (1 mm per grid), the twisting motion was recorded by a digital camera, and the data of twist angle would acquired by an image processing system. Before the measurements, the surface of the IPMC actuator was cleaned completely with clean papers.

**Table 2:** Instruments used in the test.

Name	Model	Manufacturer
Digital camera	SONY Alpha 6400	Shenzhen Sinico Optical Instrument Co., Ltd
Waveform generator	Agilent 33522A	Shenzhen Junhuang Technology Co., Ltd
Power amplification	–	Chao Hu University

## Result and discussion

Three IPMCs with different electrode separations were fabricated. The size of each IPMC was 30 mm × 29 mm. The electrode separation of the three IPMCs was 3 mm, 5 mm, and 7 mm respectively. The edges of the IPMCs were cut off to avoid causing a short circuit.

In the twist angle test, the end of IPMC was clamped by two electrode clamps. The driving voltage was the sinusoidal voltage generated by the waveform generator, with the amplitude varied from 3 to 5 V and the frequency varied from 0.1 to 0.3 Hz.

**Figure 3:**
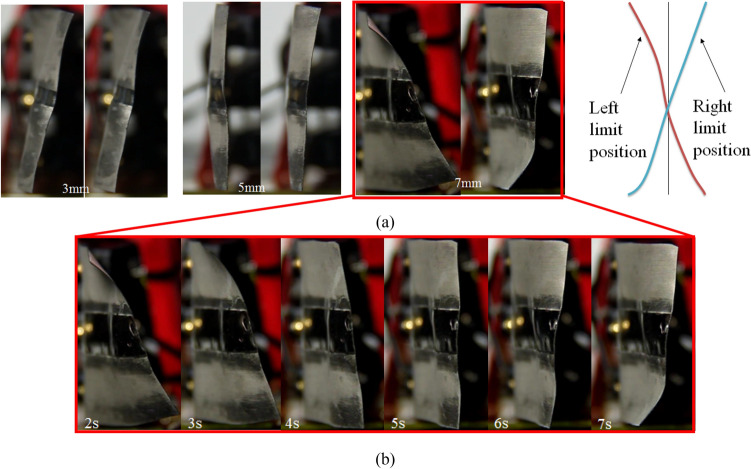
The limit position of IPMCs under AC voltage drive. (**a**) Different electrode separation under 3 V 0.1 Hz. (**b**) Twisting motion at different moments.

Figure 3a shows the state of the left and right limit positions of the IPMCs driven by an AC voltage with electrode separations are 3 mm, 5 mm, and 7 mm. The twisting motion of the IPMC with electrode separation of 7 mm from the left limit position to the right limit position is shown in Fig. 3b. Obviously the IPMC with electrode separation of 7 mm exhibits a better twisting motion.

**Figure 4:**
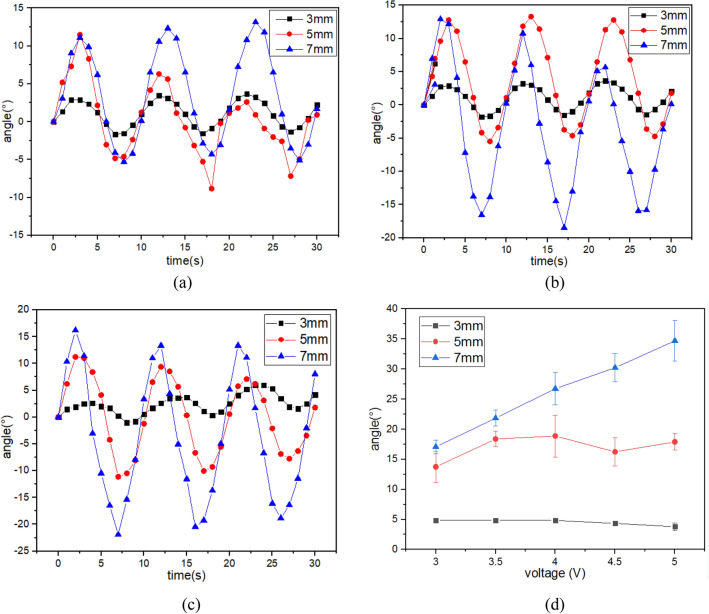
The twist angle of IPMC with patterned electrodes under 0.1 Hz AC Voltage. (**a**) 3v, (**b**) 4v (**c**), 5v (**d**) peak-valley angle under different voltages.

**Figure 5:**
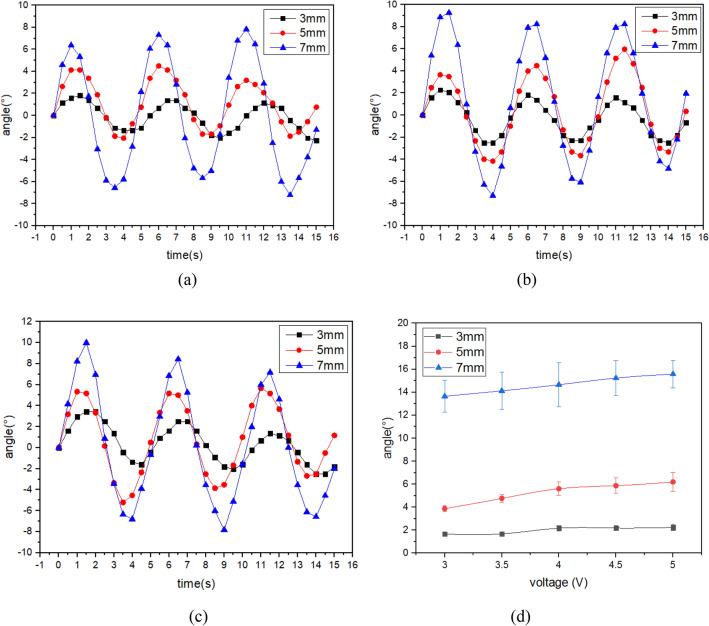
The twist angle of IPMC with patterned electrodes under 0.2 Hz AC Voltage. (**a**) 3v, (**b**) 4v, (**c**) 5v, (**d**) peak-valley angle under different voltages.

**Figure 6:**
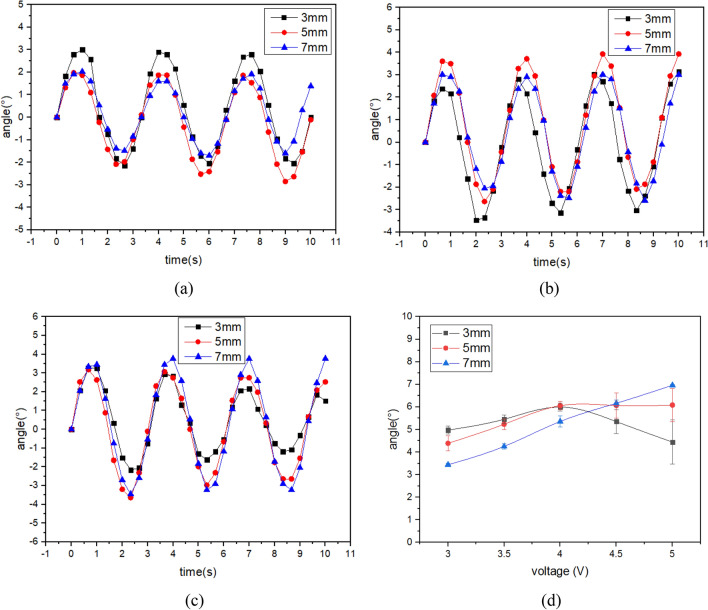
The twist angle of IPMC with patterned electrodes under 0.3 Hz AC Voltage. (**a**) 3v, (**b**) 4v, (**c**) 5v, (**d**) peak-valley angle under different voltages.

Figure 4 shows the results of the torsion performance test under 0.1 Hz AC voltage. The twist angles change periodically and are basically consistent with the sinusoidal drive voltage. The peak and valley angles are asymmetric, which is caused by the uneven distribution of hydrated cations and the influence of the manufacturing process. Similarly, you can see the results in Figs. 5 and 6. It is not accurate to express the twist angle with the peak or valley angle. In the following sections, the twist angle of IPMC represents the peak-valley angle.

Figure 4d shows that as the voltage increases, the twist angle of the IPMC with patterned electrodes increases. Evidently, as the electrode separation increases, the twist angle of the IPMC increases gradually. The result in the Fig. 4d shows that the twist angle of the IPMC with an electrode separation of 7 mm can reach 38° at 0.1 Hz with amplitude 5 V, which is two times larger than the twist angle generated by the IPMC with an electrode separation of 5 mm and six times larger than the twist angle generated by the IPMC with an electrode separation of 3 mm. However, the IPMC with electrode separation of 3 mm does not change significantly, and the twist angle is close to 5°. The IPMC with an electrode separation of 7 mm shows better torsion performance than other IPMCs. As the electrode separation increases, the amount of platinum on the IPMC surface decreases. Therefore, the smaller electrode separation of IPMC has a greater stiffness which results in a smaller deformability and twist angle.

Figure 5d shows the result of the twist angle at 0.2 Hz AC voltage. The conclusion is the same as that in Fig. 4d. Compared with the twist angle of IPMCs driven by the 0.1 Hz AC voltage, the twist angle of Fig. 5 is much reduced.

Figure 6 shows the twist angle of IPMCs at 3 Hz AC voltage. As can be seen at these higher driving frequencies the twist angle no longer changes with increasing electrode separation. Only the twist angle of the IPMC with electrode separation of 7 mm increased linearly. The IPMC with electrode separation of 3 mm began to decrease at 4 V, which is caused by the relaxation back phenomenon. At these higher driving frequencies, the hydrated cations with water molecules move quickly and the water molecules in the membrane do not have enough time to reach the other side of IPMC, resulting in reduced deformation of IPMC. It can be seen from Figs. 4, 5 and 6 that the twist angle of IPMCs decreases with increasing frequency.

## Conclusions

In this paper, we proposed a method to optimize the torsion performance of IPMCs by changing the electrode separation, which is of great importance to develop bionic mechanical systems. In fact, it can further optimize the torsion performance of IPMCs prepared by any method. Three IPMCs with patterned electrode were fabricated. The size of each electrode was 29 mm × 30 mm, and the electrode separations were 3 mm, 5 mm and 7 mm, respectively. The experiment of twisting motion was carried out to verify how the performance changes in practice. The results show that the torsion performance is improved with the electrode separation increased. The smaller electrode separation of IPMC has high stiffness which results in a smaller deformability and twist angle, while the wider electrode separation of IPMC has low stiffness and a higher deformability. The optimum electrode separation and test frequency are not discussed in this paper. However, those factors will be further investigated in the field of biomimetic actuators and sensors.
